# Transcatheter Mitral Valve Implantation in Failed Transventricular Mitral Valve Repair

**DOI:** 10.1016/j.jaccas.2024.102273

**Published:** 2024-02-26

**Authors:** Giulia Agostini, Alessandro Vairo, Antonio Montefusco, Matteo Marro, Andrea Costamagna, Michele William La Torre, Anna Chiara Trompeo, Marco Pocar, Mauro Rinaldi, Stefano Salizzoni

**Affiliations:** a Cardiac Surgery, Department of Surgical Sciences, University of Turin, Turin, Italy; b Cardiac Division, Città della Salute e della Scienza, Turin, Italy; c Cardiac Surgery, Città della Salute e della Scienza, Turin, Italy; d Anesthesiology and Intensive Care Division, Department of Surgical Sciences, University of Turin, Turin, Italy; e Cardiovascular Anesthesia and Intensive Care Division, Città della Salute e della Scienza, Turin, Italy

**Keywords:** mitral regurgitation, TMVI, transcatheter mitral valve implantation, transventricular mitral valve repair

## Abstract

An 84-year-old man presented with dyspnea at rest due to severe mitral regurgitation. He first underwent transventricular mitral valve repair with the Harpoon system, which relapsed owing to rupture of neochords. He was definitively treated with transcatheter mitral valve implantation of the Tendyne system 8 months later.

## Graphical abstract

**Figure:**
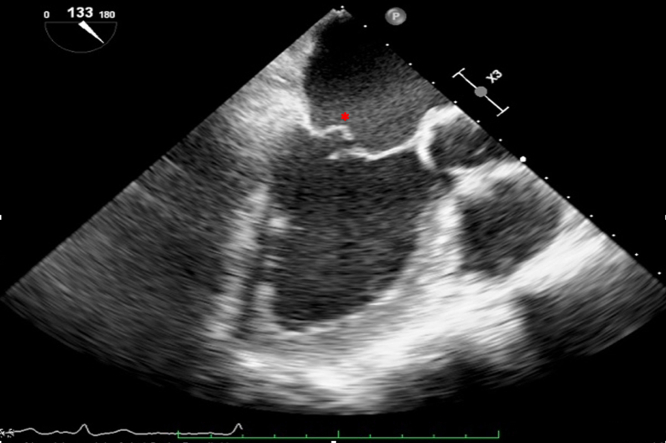

## History of Presentation

An 84-year-old man came to our attention for angina and dyspnea at rest for about 3 years, NYHA functional class IV. The symptoms were related to severe mitral regurgitation (MR) due to prolapse and flail of P2 due to chordal rupture first detected in January 2021 and not related to the chronic ischemic event. Medications included beta-blocker, angiotensin-converting enzyme inhibitor, diuretic agents, acetylsalicylic acid, and apixaban. His blood pressure was 100/70 mm Hg. He was in sinus rhythm, with a history of paroxysmal atrial fibrillation.

**Learning Objectives**

• To consider TMVI a safer approach in patients with severe MR and important comorbidities.
• To consider TMVI as a solution for patients with recurrent mitral regurgitation after TMVR.
• To consider a second transapical access possible after the first attempt failed.

## Past Medical History

Comorbidities included hypertension, mild renal failure, and right carotid endarterectomy. In February 2021 he was hospitalized for acute pulmonary edema, and during coronary angiography he underwent percutaneous transluminal coronary angioplasty and drug-eluting stent (Biofreedom) placement for critical coronary disease of the left intraventricular coronary artery and right coronary artery.

## Differential Diagnosis

The patient’s symptoms (angina and dyspnea at rest) could be associated with ischemic heart disease and severe tricuspid regurgitation, but there was no improvement after the percutaneous transluminal coronary angioplasty with drug-eluting stent placement and optimization of diuretic therapy, so those were closely related to the sudden worsening of MR.

## Investigations

**Figure 1:**
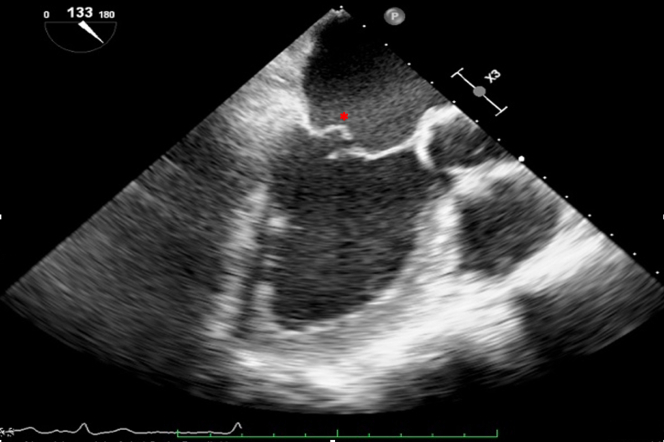
Preoperative Transesophageal Echocardiography Biplane View A cut plane through the medial (A3-P3) part of the valve showing prolapsing posterior mitral valve leaflet tips (red asterisk).

**Figure 2:**
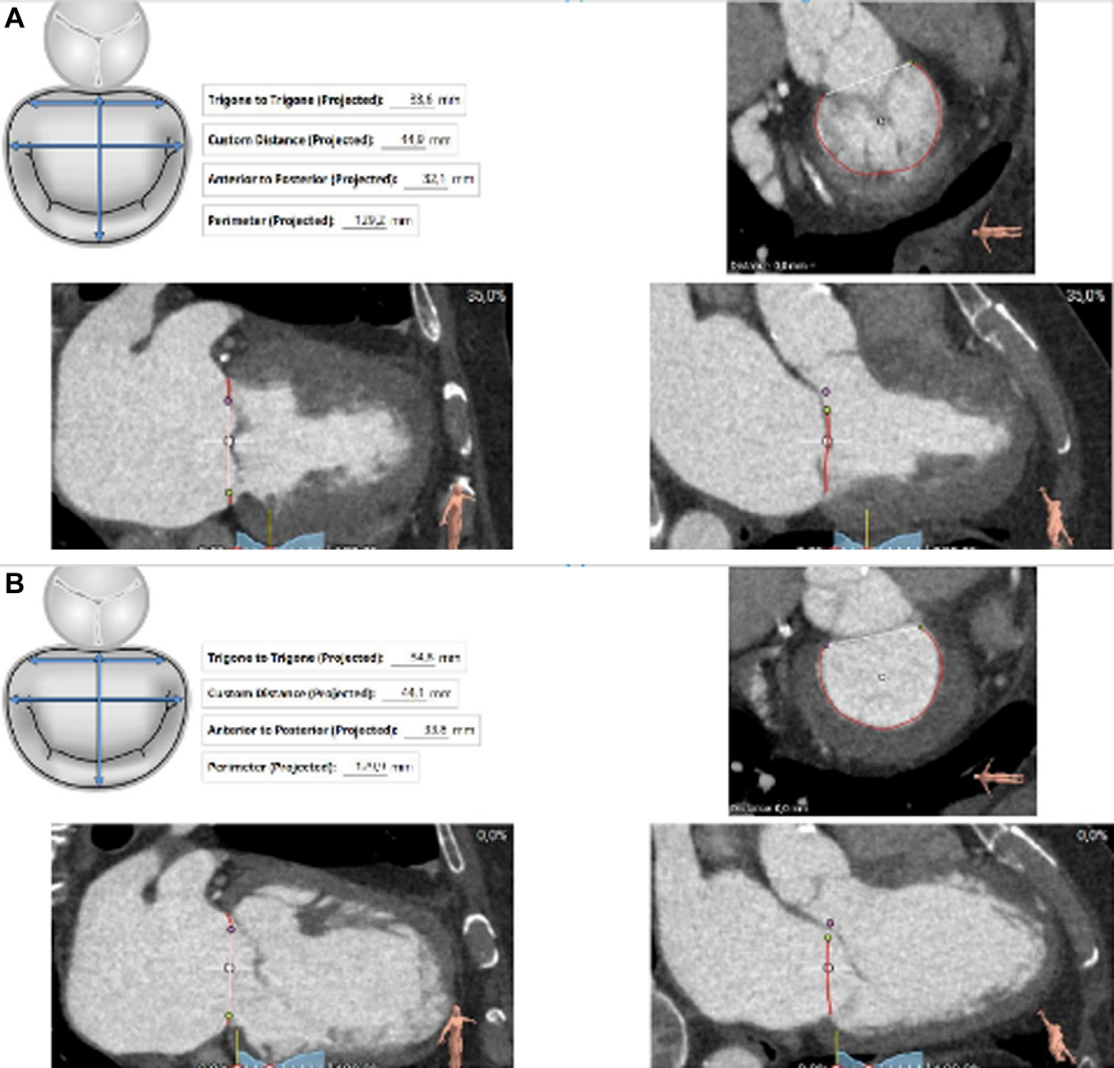
Cardiac Computed Tomography Study of annular segmentation during (A) systole and (B) diastole.

**Figure 3:**
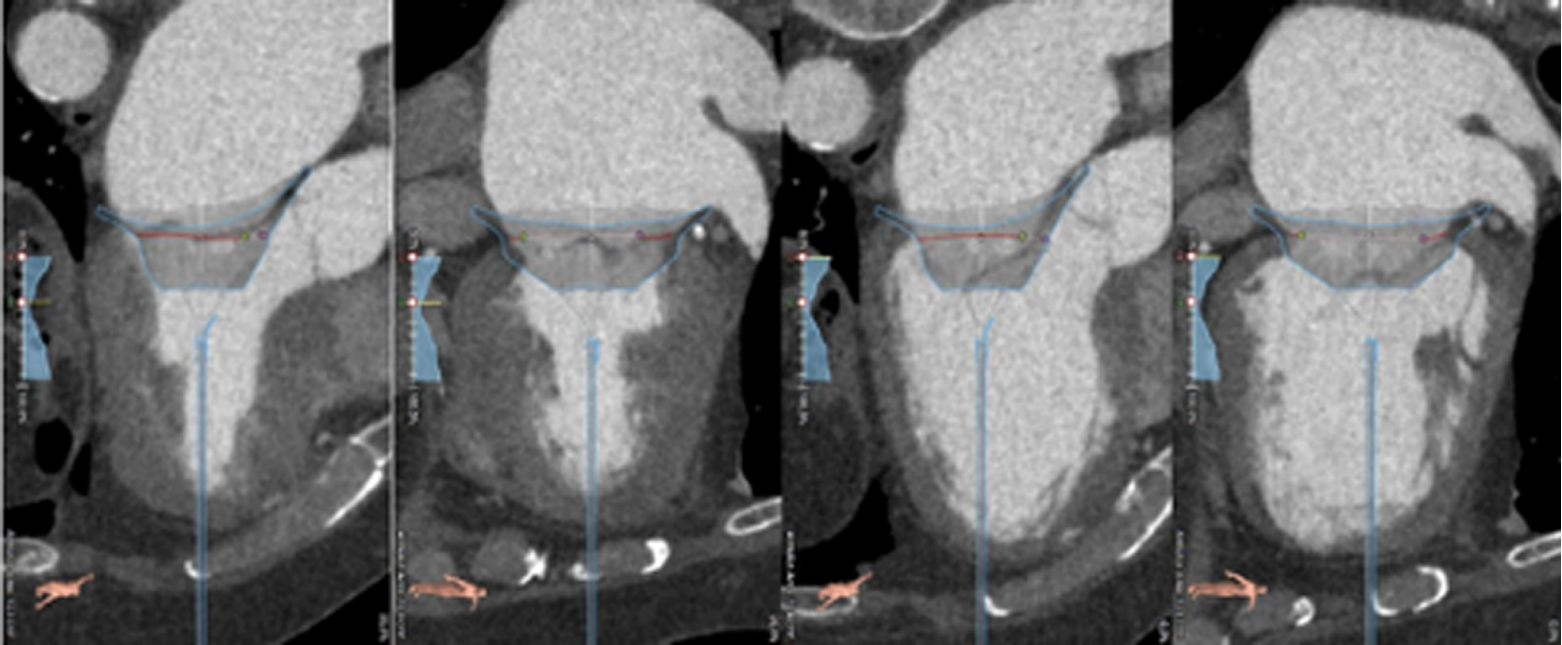
Cardiac Computed Tomography Radiologic study of the position of the Valve 37M LP during systole and diastole.

**Figure 4:**
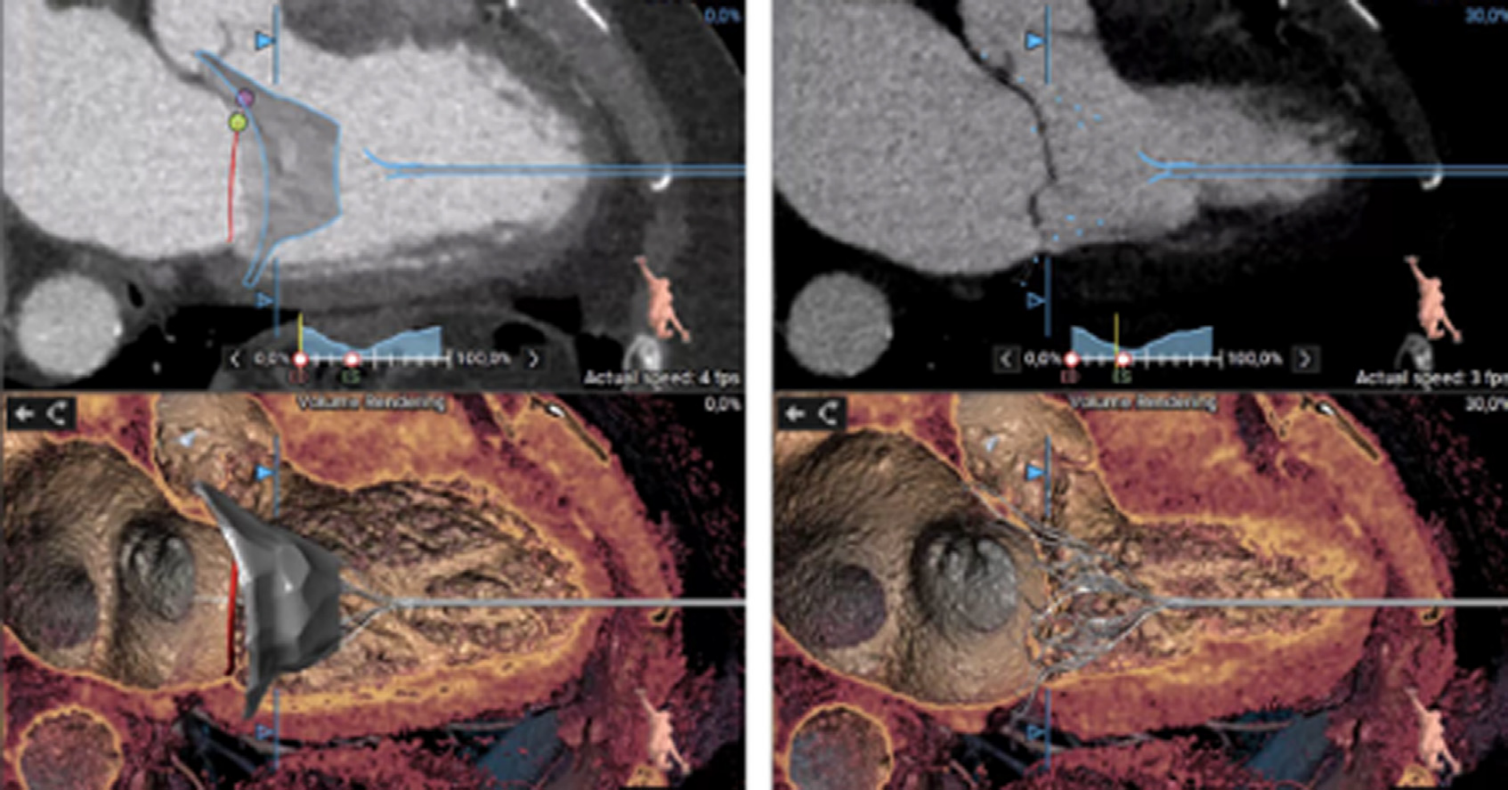
Cine Cardiac Computed Tomography Three-dimensional reconstruction of the position of the Valve 37M LP during systole and diastole.

Transthoracic echocardiography was performed in January 2021 and showed severe MR due to posterior mitral leaflet prolapse (P2) and flail due to chordal rupture (confirmed on the next transesophageal echocardiography [TEE]) (Figure 1, Video 1). Left ventricular ejection fraction was preserved. In March 2022, heart computed tomography showed feasibility for transcatheter mitral valve repair (TMVR) (Figure 2, Figure 3, Figure 4).

## Management

Owing to the high-risk profile and severe symptomatology, a TMVR procedure with the Harpoon system was approved by the heart team. A mitral transcatheter edge-to-edge repair was not chosen, to avoid a fixed coaptation point unfavorable in case of a reoperation. Advanced age, peripheral vascular disease, pulmonary hypertension, and ischemic heart disease are some of the reasons that determine high risk of mortality and morbidity (EuroSCORE II 9.10%, Society of Thoracic Surgeons score 8.60%). The patient was thoroughly informed of the procedure and signed a specific consent.^1^

In November 2021 the procedure was performed with TEE guidance and the patient under general anesthesia. Through a left minithoracotomy and an anterior left access, the plunger was pushed and the P2 prolapsing segment was punctured with a needle wrapped with a coil of expanded polyfluoroethylene. Three chords were released. After tensioning there was no residual MR.

**Figure 5:**
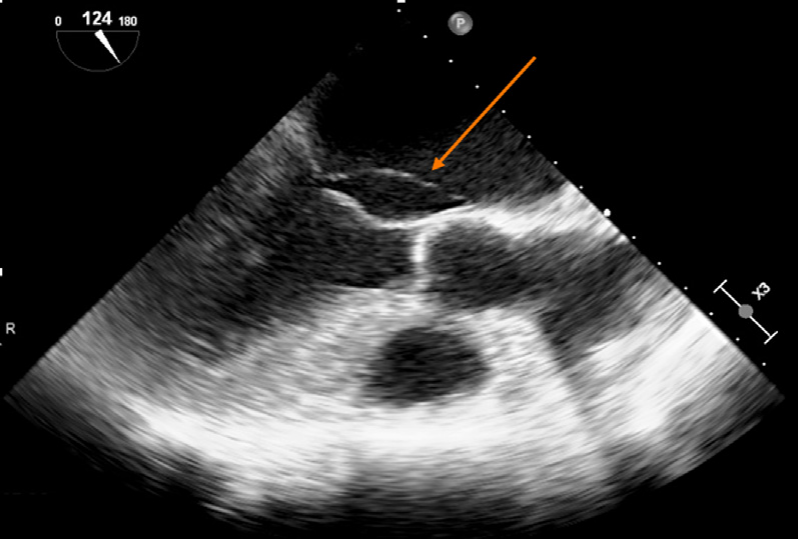
Failed Harpoon Procedure Rupture of a chord and flail (indicated by red arrow) at the ventricular apex, leading to severe failure with eccentric jet.

The patient returned because of sudden dyspnea after 3 months. In April 2021, echocardiography revealed severe MR due to chordal rupture. The causes could be ventricular laxity in an ischemic area or chordal elongation which determine their rupture (Figure 5, Video 2).

**Figure 6:**
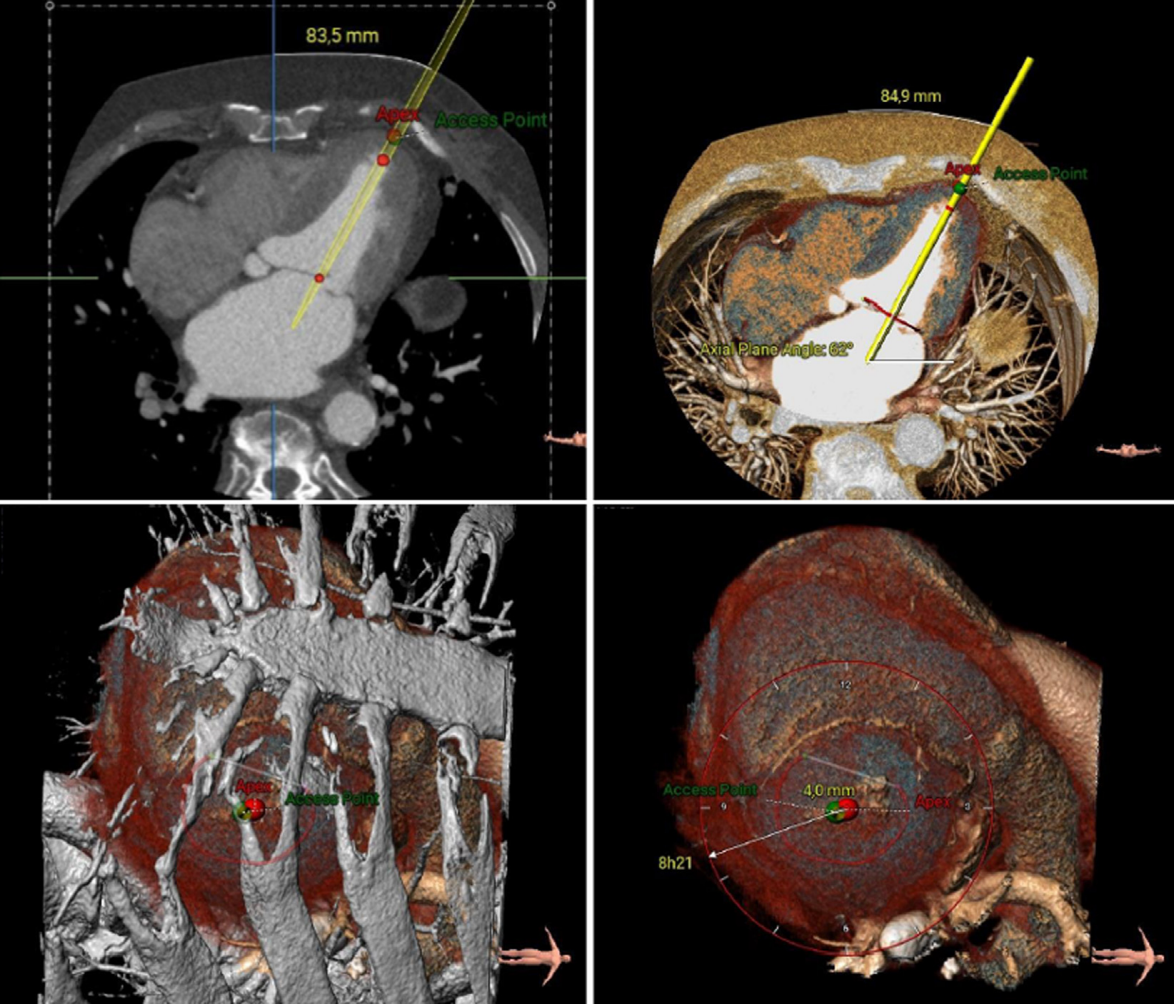
Preoperative study of Thoracic Access and Apical View Red = apex; green = target access site.

**Figure 7:**
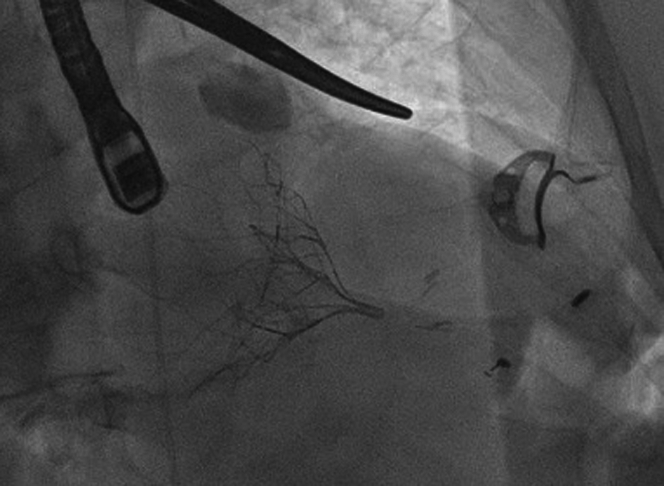
Tendyne Procedure The prosthesis released by the Tendyne LP 37M system is visible in the left ventricle.

In view of the patient’s clinical conditions and quality of life, the heart team deemed him to be suitable for transcatheter mitral valve implantation (TMVI) with the Tendyne bioprosthesis. In July 2022, the patient underwent implantation of the Tendyne LP 37M via left minithoracotomy under general anesthesia. Through the left ventricle apex (Figure 6) and under TEE guidance, the TMVI was released and fixed to the entry site with the use of a large pad. At the final TEE, the bioprosthesis was normally positioned, gradient was 2 mm Hg, and no paravalvular leak or left ventricular outflow tract obstruction was seen (Figure 7). The procedure lasted 210 minutes. Postoperative course was uneventful, and the patient was discharged home on the eighth postoperative day.

## Discussion

Conventional surgical repair is the criterion standard for MV prolapse (Carpentier Type II). The off-pump TMVR with expanded polyfluoroethylene chordal insertion using the Harpoon TSD-5 device (Edwards Lifesciences) seemed a valid alternative.^2^ Echocardiographic features for patient selection consist of MR due to P2 prolapse and sufficient coaptation measured by ratio of the tissue (length of the prolapsing segment) to the gap (distance between the free edge of the anterior mitral valve leaflet and the base of the posterior mitral valve leaflet) (ideally >1.5).^1^ In the present case, the coaptation was sufficient: A minimum tissue/gap ratio of 1.5:1 was measured.

This case proves that a patient subjected to a previous procedure via left transventricular access can still be subjected to a second access after a careful study of the case, the cardiac contractility, and the free wall of the left ventricle. Cases have already been described in which it was possible to perform a second TMVR using a NeoChord DS 1000 (NeoChord) through a second and different transapical access, without the need to convert to traditional surgery. The difficulty is the approach through a weak myocardium subjected to previous transapical access in a chronically ischemic area. Therefore, it is necessary to plan the entry area of the device through radiologic studies and then proceed with the surgical procedure under TEE guidance and “finger tests” (Figure 4). The risks of a second transapical access are related to the fact that pseudoaneurysms could develop because of the potential loss of function of the left ventricle as well as bleeding.^3^

In the face of severe MR due to chordal rupture, TMVI was considered to be the safest approach.

Tendyne is now the only CE-marked TMVI. It is a self-expanding trileaflet porcine pericardial valve composed of 2 self-expanding nitinol stents.^4^^,^^5^ It is fully repositionable and retrievable, with a survival rate of 96%.^6^ Anticoagulant therapy is necessary for 3 to 6 months (target international normalized ratio 2.5), but the risk of thrombosis is low and then life-long low-dose aspirin is reasonable for elderly patients.

## Follow-Up

At the 6-month echocardiographic follow-up, the patient was in NYHA functional class I-II. On echocardiography, the medium gradient was 6 mm Hg with minimal paraprosthetic leak in the medial site (Video 3). The patient did not report dyspnea and had resumed his daily activities with an improvement in his quality of life.

## Conclusions

This case represents the first reported successful TMVI in failed TMVR and proves that it can be considered as a safe solution for patients not eligible for traditional surgery.

## Funding Support and Author Disclosures

The authors have reported that they have no relationships relevant to the contents of this paper to disclose.

## Appendix

Video 1 Preoperative Transesophageal Echocardiography Biplane View A cut plane through the medial (A3-P3) part of the valve showing prolapsing posterior mitral valve leaflet tips.

Video 2 Transesophageal Echocardiography Shows the Failure of the Harpoon Procedure Rupture of a chord and flail at the ventricular apex.

Video 3 Postoperative Echocardiography An acceleration of the anterograde flowmetry (mean gradient of 6 mm Hg) and a minimal paraprosthetic leak in the medial site.
